# Towards a post‐COVID world: Challenges and progress of recovery in Kazakhstan

**DOI:** 10.1002/puh2.17

**Published:** 2022-09-02

**Authors:** Usman Abubakar Haruna, Oladunni Abimbola Amos, Dawa Gyeltshen, Paolo Colet, Joseph Almazan, Attaullah Ahmadi, Antonio Sarria‐Santamera

**Affiliations:** ^1^ Department of Medicine Nazarbayev University School of Medicine (NUSOM) Nursultan Kazakhstan; ^2^ Department of Pharmacology and Therapeutics Faculty of Pharmaceutical Sciences Ahmadu Bello University Zaria Nigeria; ^3^ Department of Pharmacology and Therapeutics University of Ibadan, Ibadan, Oyo State Nigeria; ^4^ Afe Babalola Multi‐System Hospital Ado‐Ekiti Nigeria; ^5^ Department of Medicine Jigme Dorji Wangchuk National Referral Hospital Thimphu Bhutan; ^6^ Medical Research Center Kateb University Kabul Afghanistan

**Keywords:** challenges, COVID‐19, herd immunity, Kazakhstan, pandemic, vaccination

## Abstract

Kazakhstan announced the first cases of COVID‐19 in March 2020. Within a span of a few months, the pandemic ravaged all regions affecting vulnerable populations due to limited access to healthcare services and co‐morbidities. To minimize the spread of the pandemic, the government announced the implementation of containment measures such as quarantine, movement restrictions, and lockdowns among others. The collateral effect of the pandemic has disrupted economic and learning activities pushing several people below the poverty line. The pandemic revealed the weakness of healthcare including the acute shortage of essential medicines and lack of hospital beds. This calls for stringent measures to revive the economy and mitigate the reeling effect of the pandemic. As a result, Kazakhstan commenced COVID‐19 vaccination efforts in February 2021. To date, about 47.8% are fully vaccinated pushing Kazakhstan closer to achieving herd immunity at the 60% threshold. However, the country faces challenges such as vaccine hesitancy and uncertainty surrounding vaccine effectiveness against new variants of SARS‐CoV2, among others. This paper aims to explore the health and socioeconomic challenges caused by COVID‐19 in Kazakhstan, control strategies, vaccination campaigns and progress towards herd immunity.

## Graphical abstract

This paper describes the COVID‐19 situation in Kazakhstan with special focus on the country's vaccination efforts against COVID‐19. It also explores the challenges and progress the country has made thus far in regard to COVID‐19 vaccination.

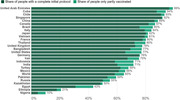

## INTRODUCTION

The first cases of COVID‐19 in Kazakhstan were reported on 13 March 2020, following the arrival of two Kazakh citizens from Germany [1], within a span of a few months, the virus spread to all regions across the country. The rapid spread of the virus triggered the government of Kazakhstan to develop and implement containment measures, such as, quarantine, declaration of a state of emergency, closure of schools including non‐essential businesses, and movement restrictions in public places. Subsequently, an initial lockdown was imposed from 16 March to 11 May 2020, followed by a series of lock‐downs which ended on 16 August 2020. The severe rise of new cases at the end of June and the beginning of July prompted the government to introduce the second quarantine on 5 July 2020. A gradual easing of the second quarantine was started on 17 August 2020, but a weekend lockdown was kept till the end of August [2].

These measures in turn created a major socioeconomic consequence that disproportionately affected vulnerable populations, as well as Small and Medium Enterprises (SMEs) that provided jobs, incomes, and tax revenue that help fund government services [3]. In April 2021, the mobile application ‘Ashyq’ was launched in Kazakhstan to ensure that businesses continue their operations during the quarantine period, minimize the spread of COVID‐19, and reduce the number of contacts leading to transmission [2]. Through the use of a QR code and integration with the general database of the Ministry of Health of Kazakhstan, Ashyq could determine the current viral status of an individual based on the four risk ratings from the government data: red, yellow, blue and green before being allowed to enter public settings, these colours signify the severity of infection with the virus [2].

The COVID‐19 pandemic has further exposed weaknesses in healthcare systems, such as health policies, medical supply chains, healthcare workforce, access to quality healthcare and pandemic preparedness [4]. The national government through the support of the Kazakhstan WHO Country Office responded well to the COVID‐19 outbreak by implementing several measures in an attempt to control the spread of the virus. These measures include raising for resource allocation, recruitment and specialized staff training, an effective risk communication system, and the development of contextualized guidelines. These approaches led to efficient incidence and resource management including knowledge generation [5].

**FIGURE 1:**
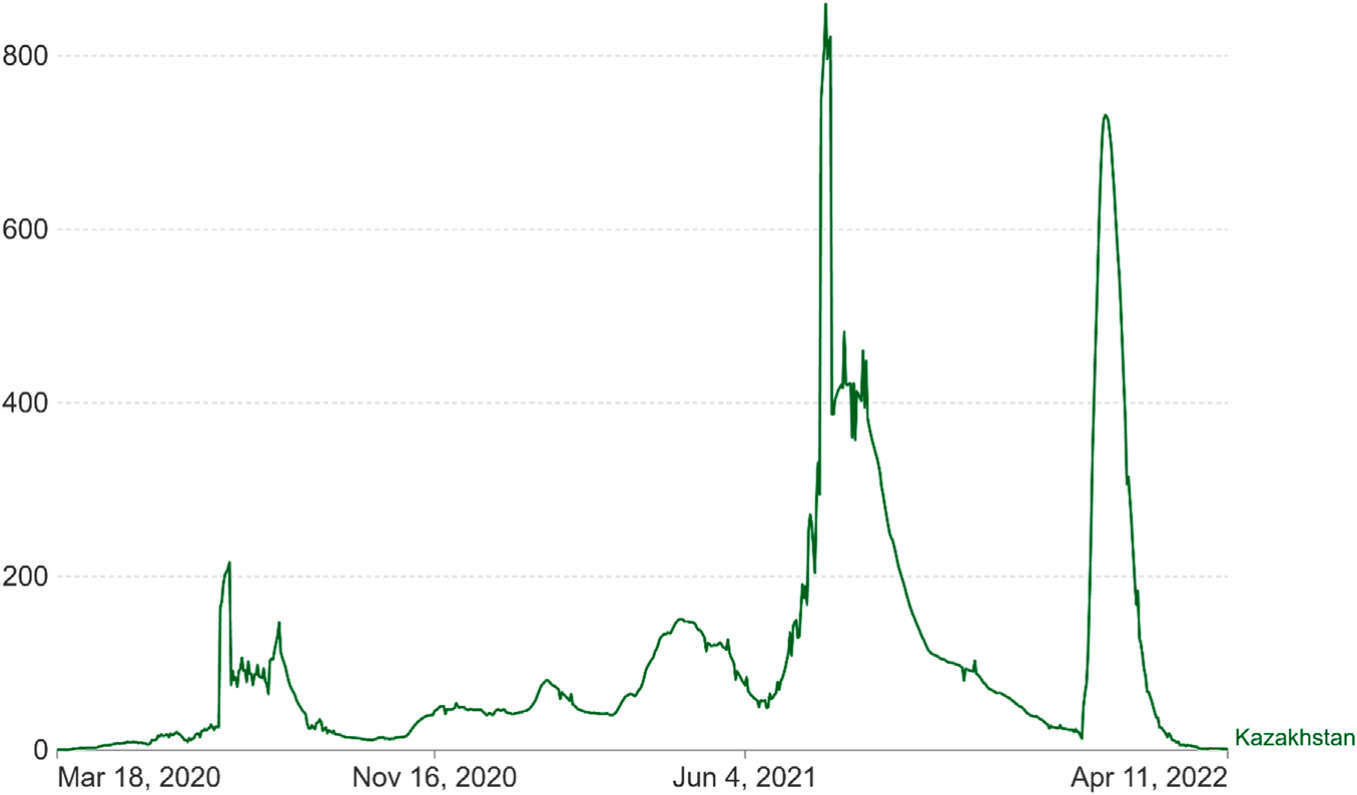
Number of COVID‐19 cases in Kazakhstan (*Source*: John Hopkins University CSSE COVID‐19 Data).

As of 12 April 2022, there are over 500 million confirmed cases and 6 million deaths reported due to COVID‐19 pandemic, globally [2]. Kazakhstan with a population of 18.63 million and a population density of six people per square kilometre is one of the 223 countries affected by COVID‐19 worldwide [6]. Since, the announcement of the first two cases of COVID‐19 in Kazakhstan, the virus has continued to spread across the country with over 1,394,093 million confirmed cases and 19,013 deaths as can be seen in the Figure 1 [7]. These statistics reflect the speed and degree with which the pandemic has suffered different waves related to the diverse variants that have circulated globally. The very reduced viral sequencing in the country seriously limits the determination of exactly the predominant variants. The last significant wave occurred between January and February most likely driven by the Omicron variant.

Two reflections are necessary when assessing the magnitude of the pandemic. One is a problem affecting all countries: despite the significant effort of public health authorities to identify all cases and contacts, and the extended testing capabilities in Kazakhstan, official case reporting may undercount the number of infections by at least 60% [8]. A significant proportion of asymptomatic cases that have remained undocumented may have contributed to the extension of the pandemic. Another is more specific to Kazakhstan: the existence of a significant proportion of atypical pneumonia of unknown origin with clinical symptoms, computed tomography findings, and epidemiological characteristics similar to COVID‐19, but negative PCR results, which were later defined as ‘COVID‐19‐like pneumonia’ [9]. The last official statistics reflect more than 88,000 cases of ‘COVID‐19‐like pneumonia’. This paper aims to explore the health and socioeconomic challenges caused by COVID‐19 in Kazakhstan, control strategies, vaccination campaigns and progress towards herd immunity.

As the COVID‐19 pandemic began to take a downward trajectory in Kazakhstan in the last weeks of 2021, the Omicron variant coupled with the protest that occurred in January caused a spike in infections that was sustained through February 2022. The wave of protests that broke out in Kazakhstan on 2 January 2022 was in part due to the sudden rise in prices of liquefied gas after the removal of a government‐imposed price cap on 1 January [10]. The peaceful demonstrations began in the oil‐producing city of Zhanaozen, which spread quickly to other cities, including the nation's largest, Almaty, where the demonstrations turned violent amid growing dissatisfaction with the regime and rising economic inequality [11, 12]. This protest resulted in a decline of vaccination uptake [13], which could threaten the country's gain in the fight against the notorious COVID‐19 and increased the surge of new infections, a development that resulted in the declaration of another state of emergency and enforcement of curfew, this was an attempt to restore peace and contain the spread of the COVID‐19 pandemic.

Since, the Government announced the identification of Omicron in Kazakhstan, the same pattern has been observed in other countries where Omicron has been reported to also place: an extraordinarily rapid growth followed by an abrupt decline. Interestingly, in Kazakhstan, the Omicron wave peaked in just 2 weeks and since then we have observed a continuous decrease in cases. Since, the 1st week of March, the country reports less than 100 daily cases. No effect was observed after Nawroz, the Central Asian celebration of the ‘New Year’, which is celebrated for several days starting from the 21st of March, the day of the spring equinox, and that is the most important holiday in the Kazakh calendar. Since, April the daily reported cases are less than 20. This low number of cases has been accompanied with a parallel reduction in hospital admissions and cases being treated as outpatients. As a consequence, the daily number of active COVID‐19 cases in Kazakhstan is less than 200. The explanation for this significant descent in the number of cases and the stability we continue to observe, most likely due to the ‘immune wall’ in the country, either from previous infections or vaccination is not clear. Given the very low number of cases the Government has eliminated all restrictions and measures aimed to control the spread of the SARS‐CoV‐2.

## IMPACT OF COVID‐19 ON EDUCATION AND HEALTHCARE SYSTEM

Two years into the pandemic, the world is still suffering the consequences, with the education sector one of the hardest hits. At the peak of the pandemic lockdown, 90 percent of students worldwide suffered school closures, leaving more than a third without access to remote learning. For Kazakhstan, COVID‐19 has been a challenge for the education sector which was already struggling even before the pandemic, the shift from offline to online formats of learning, has left many students, kids and parents struggling to adjust to the new reality [12]. Consequently, it has become increasingly important to use E‐Learning platforms to effectively engage learners: however, the unanticipated transition to mass digitalization has revealed the inadequacies of the education system, unequal access to electronic learning equipment, poor internet speed, and the unpreparedness of the people involved [12, 14].

Furthermore, the online videos, digital content, and discussion forums cannot succeed in providing a holistic teaching‐learning outcome, since learning outcomes are not merely measured by the level of grade or amount of knowledge acquired [14]. Another problem posed by this is a decline in class attendance, poor performance and increased cheating among students and the lack of preparation of the people involved [14]. UNICEF, in partnership with UNESCO and the Ministry of Education and Science of Kazakhstan, organized a series of webinars during the quarantine period, with about 3000 staff on digital literacy and infection control strategies [15]. In September 2020, UNICEF provided the rural remote schools in Kazakhstan with sanitizers and thermometers, in addition to information and educational materials to aid the safe reopening of schools [15].

COVID‐19 is a global healthcare crisis with unprecedented impacts on healthcare delivery. In the midst of this crisis, easy access to healthcare services is more important than ever [16]. A key finding of COVID‐19 was that health systems must be well prepared to ensure that access to essential health services is not compromised [5]. Unfortunately, the pandemic spread to Kazakhstan at a time when residents in the country were already facing limited healthcare funding, high levels of chronic disease, and limited access to health care [17]. This further compounded the impact of COVID‐19 on healthcare delivery pushing Kazakhstan to have an acute shortage of essential medicines and lack of hospital beds [18]. Within 13 days, the government constructed a brand‐new hospital in Nur‐Sultan for COVID‐19‐infected patients, a feat matched by what only China had achieved during the pandemic [19]. Given the speed with which the virus spread and the continuous demand for medicines and medical supplies, the European Union (EU) in July 2020 launched a comprehensive EUR 3 million Central Asia Crisis Response solidarity package, with a specific focus on Kazakhstan. In December 2020, the EU, in cooperation with UNICEF, had flown over 300,000 dexamethasone ampoules and 2000 pulse oximeters to Kazakhstan to support the government's effort to combat the COVID‐19 pandemic [20].

## IMPACT OF COVID‐19 ON ECONOMY AND RECOVERY PLAN

COVID‐19 represents the greatest challenge to the global economy since the Second World War. Kazakhstan, which produces about 90 million tons of oil annually, also experienced lower revenues due to declining oil prices caused by COVID‐19 [21]. Also, the lockdown measures imposed in Kazakhstan resulted in a 40% decline in domestic consumption. It is estimated that 300,000 SMEs have ceased operations nationwide, with about 1.5 million citizens on either unpaid leave or have lost their jobs [4]. The cumulative impact of the pandemic resulted in the contraction of GDP by about 2.8 percent pushing an additional number of individuals down the poverty line [21]. The Kazakh government quickly swung into action by implementing an anti‐crisis package worth $10 billion to augment the social safety net and support businesses in the face of the crisis [21]. The package also includes a tax waiver, a temporary reduction of Value Added Tax (VAT), and customs duties in addition to a concessional loan provided by the National bank [19]. Also, UNICEF jointly with its partners, including the EU, supports the most vulnerable populations and families with children in key areas such as education, child protection, and health. Also, the UNDP has launched a micro‐loan programme, which aims to support SMEs to thrive, through the programme, loans are allocated to businesses to help them cope with COVID‐19 crises [22].

## VACCINE ROLLOUT

Vaccines are both a right and a way to reopen societies and stabilize economies [23]. As many effective COVID‐19 vaccines are being rolled out globally, there is hope that herd immunity can be achieved through mass vaccination and the widespread COVID‐19 can finally be quelled or at least slowed [24]. COVID‐19 vaccination activities in Kazakhstan began in February 2021 following the delivery of 2 million doses of Russia's Sputnik V vaccine with additional 4 million doses being requested to vaccinate the eligible population [25]. In compliance with the WHO recommendations, the government of Kazakhstan mapped out a priority group that will receive the vaccine jabs, this included healthcare personnel, teachers, and employees of law enforcement agencies among others. Also, additional 1 million doses of Chinese‐developed Sinopharm vaccine were procured through the Central Asian republics’ sovereign wealth fund. These vaccine deliveries were possible due to the strong diplomatic relationship between these countries and Kazakhstan.

In April 2021, the country began the administration of its home‐grown COVID‐19 vaccine called the QazVac vaccine which was developed by the Research Institute of Biological Safety Problem. While this feat has put Kazakhstan in an elite club of pharmaceutically advanced nations, the production of the vaccine remains a bottleneck. In November 2021, the country received the first batch of Pfizer‐BioNTech vaccines which were used to vaccinate older people, children aged 12, pregnant, and breastfeeding women [25]. Other registered vaccines in Kazakhstan include Hayat‐Vax, CoronaVac and Sinopharm.

Now that vaccines are flooding the country, 49.69% of the country's population are fully vaccinated, with 50% having received at least one dose of the vaccine. This led to an increase in the population subject to vaccination from 9.9 million to 11.4 million [26]. We feel that this achievement, despite the low vaccination target, may be contributing to progress to stabilize the pandemic dynamics, even though it is unclear what proportion of the population must be vaccinated against COVID‐19 to induce herd immunity.

## PROGRESS TOWARDS HERD IMMUNITY

Kazakhstan, the richest country in central Asia, faces tremendous challenges caused by the COVID‐19 pandemic and the simultaneous plunge in oil prices, which contracted the country's economy and its ability to deal with the COVID‐19 pandemic. To recover from the reeling effect of the COVID‐19 pandemic and restore normalcy, the country implemented a multi‐pronged vaccination strategy. With the available number of vaccine candidates, many countries are now moving towards achieving herd immunity through mass vaccination to halt the spread of the virus. The term ‘herd immunity’ refers to a situation where a sufficiently large proportion of the population has immunity to a given infection such that it slows or prevents the spread of disease, protecting those considered at risk [27]. Immunity may be developed by either infection with the disease pathogen or through vaccination, which triggers the body to mount an immune response against the invading pathogens. The achievement of herd immunity will result in minimal disease transmission, and unvaccinated or unresponsive individuals who failed to develop immunity after vaccination will be protected [27]. Unfortunately, the pandemic cannot be controlled solely with vaccines, partly because new variants are more transmissible and partly because vaccines are primarily designed to protect against severe disease and death [28]. Therefore, there is a need to strengthen and sustain infection control measures.

## CHALLENGES TOWARDS ACHIEVING HERD IMMUNITY

### Vaccine hesitancy

Central Asia had already suffered from vaccine hesitancy long before the emergence of the COVID‐19 pandemic, a problem fuelled by multi‐dimensional factors such as belief, socio‐cultural and political factors. Vaccine hesitancy, a delay in accepting vaccines, or blunt refusal to be vaccinated, has become a global public health threat [15, 27]. While Kazakhstan has made huge progress in the rollout and administration of the COVID‐19 vaccine, vaccine hesitancy, including vaccination refusals, represents a major challenge to achieving herd immunity. This is fuelled by the fear associated with the safety of the vaccine. While several studies confirmed the safety of COVID‐19 vaccines, Kazakhs, however, worry that the vaccine has not yet been tested adequately to guarantee its safety. As a result, people were reported paying for fake vaccine passports, when they could get them for free, a development that could jeopardize their journey to achieving herd immunity [15]. In response, the government of Kazakhstan implemented a compulsory vaccination for employers that work in a firm of more than 20 people including weekly Reverse Transcription Polymerase Chain Reaction (RT‐PCR) test for which the cost is to be borne by individuals that refused the vaccine, a string that abates the vaccine hesitancy and increases uptake [15]. Also, the recognition of vaccine passports as a ticket to major public settings has encouraged many more people to take up the COVID‐19 vaccine. In addition, 1378 vaccination centres were established by the government to ramp up vaccinations in cities across the country.

## DURABILITY OF IMMUNITY AND BOOSTER VACCINE

**FIGURE 2:**
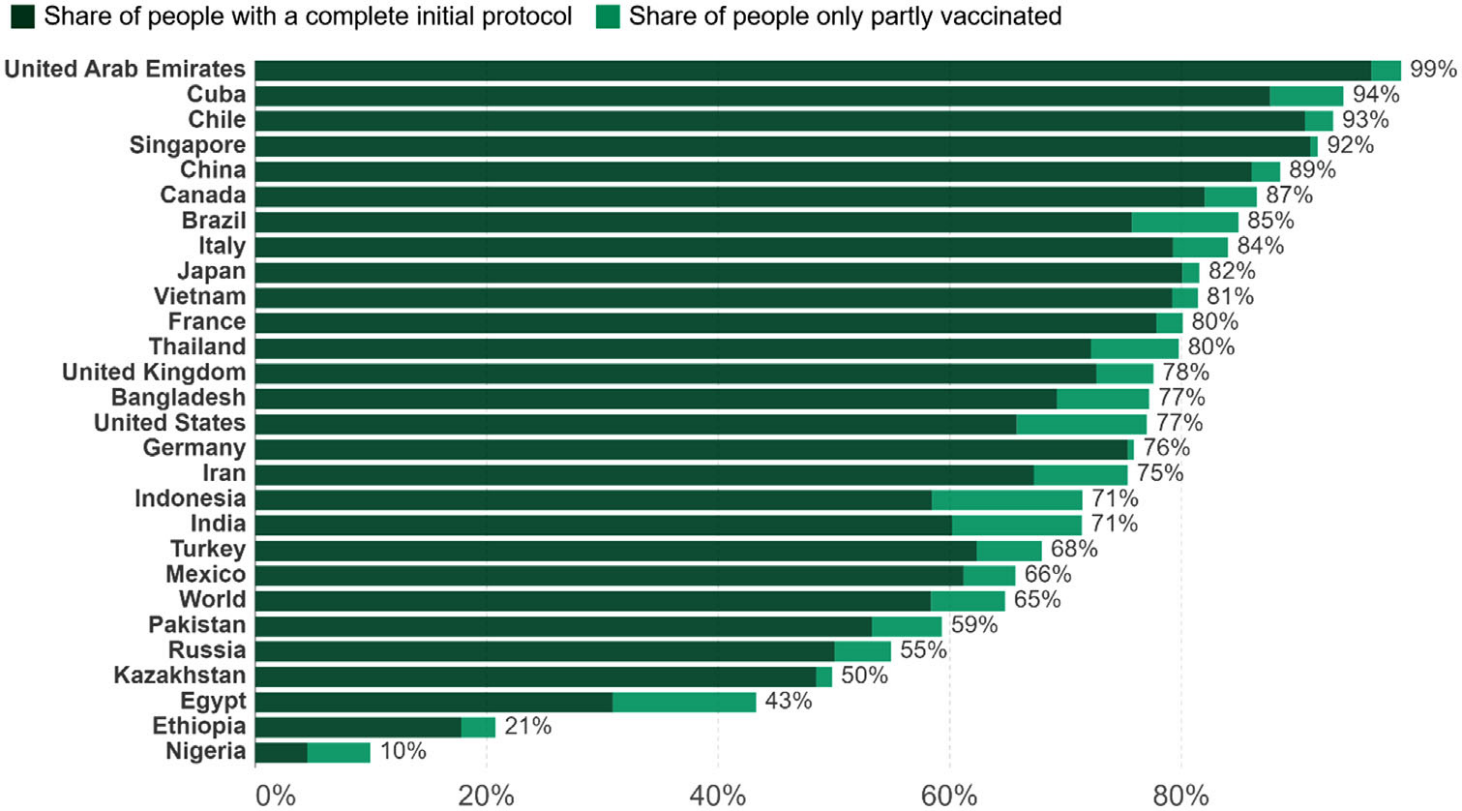
Percentage of people who received at least one dose or full vaccination in Kazakhstan (*Source*: Our World in Data).

Although, immunity is developed from natural infection or from vaccination, how long the immunity lasts remains unclear. Generally, immunity from live vaccines lasts for about 6 months, therefore, to achieve herd immunity it becomes necessary to ramp up vaccination with booster doses. To effectively begin the administration of the booster shots, the government developed guidelines to prioritize people that will receive the booster vaccine which includes teachers, medical workers, law enforcement personnel, and individuals who contracted COVID‐19 after two doses of the vaccine [24]. As a result, 13.4% of the population received booster shots as can be seen in Figure 2, therefore, there is a need for coordinated efforts to increase the uptake of booster doses so as to maintain the immunity gained. Additionally, available vaccines have proven effective in preventing the symptomatic manifestation of COVID‐19 and possibly the length of hospitalization, but we do not know whether the vaccines are effective at blocking transmission, which makes it more challenging to achieve herd immunity [29].

## VIRAL MUTATION

While countries are making efforts to ramp up COVID‐19 vaccination, the emergence and dissemination of the new strain of the virus continues to threaten to reverse the achievements gained in the fight against the virus. COVID‐19 has evolved, and new variants have emerged, the latest being the Omicron [30]. The pandemic is largely driven by the Delta strain, against which available vaccines have constantly offered a significant level of protection. Hence, there is a need for Kazakh government to continue to scale‐up vaccination coverage.

On 6 January 2022, Kazakhstan confirmed the first case of the variant. This prompted the government to enforce additional social and public health measures which involve exclusive access to shopping malls, entertainment venues, and sports facilities by people who have been vaccinated. Without immediate scaled‐up public and social measures, the sheer number of cases due to the new strain may overwhelm the health system. While research into how vaccines work against COVID‐19 variants is ongoing, viral mutation could be one factor that may hinder the attainment of herd immunity in Kazakhstan. Although, there is no evidence yet that the virus has mutated in ways that make it resistant to the effect of the vaccine, the emerging variants present with a high level of transmissibility and contagiousness [16]. There is a need for the government to review the current health system status and strengthen disease surveillance, and human resource capacity as well as intensify rapid contact tracing to mitigate the impact of new variants.

Since the onset of SARS‐CoV‐2 pandemic, there has been a wide number of people affected worldwide, with important implications not only for the infected and healthcare professionals, but also for the entire society, including economical and mental health concerns. The emergence of novel variants has represented, during the pandemic and still represents in the immediate future, important challenge to face to put an end to this long public health challenge. The intrinsic properties of SARS‐CoV‐2, including their re‐combination and genetic basis and the selective pressure exerted on this virus favours the evolution of SARS‐CoV‐2 leading to the appearance of different variants. Public health preparedness including strengthening surveillance and genetic sequencing, and vaccination remain essential pillars, as well a critical need for the development of novel vaccines against SARS‐CoV‐2 with the aim in a pan‐corona universal vaccine.

## CONCLUSION AND RECOMMENDATIONS

Kazakhstan has made tremendous strides towards controlling the COVID‐19 pandemic. Based on the available statistics of vaccinated people and effective control strategy, it can be summarized that Kazakhstan has the ability to reach herd immunity, this will help the country to reactivate the economy and recover from the reeling effect of the COVID‐19 pandemic. However, more people need to be vaccinated. Also, to gain immunity from vaccines, full recommended doses are needed, and to sustain the immunity gained, booster doses are essential. Therefore, the country needs to increase vaccination centres and sustain policies that promote vaccination uptake among citizens. Also, community mobilization initiatives through public education and social media campaigns should be implemented to dispel rumours about safety concerns and vaccine hesitancy.

## AUTHOR CONTRIBUTIONS

Usman Abubakar Haruna: Conceptualization; Writing – original draft; Writing – review & editing. Paolo Colet: Writing – review & editing. Joseph Almazan: Writing – review & editing. Attaullah Ahmadi: Supervision; Writing – review & editing. Antonio Sarria‐Santamera: Supervision; Writing – review & editing. All authors read and approved the manuscript.

## ETHICS STATEMENT

This is a commentary. There is no need for ethical approval

## Data Availability

No database or primary data was used in preparing the manuscript.
